# A Fast and Refined Cancer Regions Segmentation Framework in Whole-slide Breast Pathological Images

**DOI:** 10.1038/s41598-018-37492-9

**Published:** 2019-01-29

**Authors:** Zichao Guo, Hong Liu, Haomiao Ni, Xiangdong Wang, Mingming Su, Wei Guo, Kuansong Wang, Taijiao Jiang, Yueliang Qian

**Affiliations:** 1 Beijing Key Laboratory of Mobile Computing and Pervasive Device, Institute of Computing Technology, Chinese Academy of Sciences, Beijing, 100190 China; 2 Research Center for Big Data of Biomedical Sciences, Institute of Basic Medical Sciences, Chinese Academy of Medical Sciences & Peking Union Medical College, Beijing, 100005 China; 3 Suzhou Institute of Systems Medicine, Suzhou, 215123 China; 4 Graduate School of Peking Union Medical College, Beijing, 100005 China; 5 Department of Pathology, Xiangya Hospital, Central South University, Changsha, Hunan 410013 China; 6 Department of Pathology, School of Basic Medical Sciences, Central South University, Changsha, Hunan 410013 China

## Abstract

Supervised learning methods are commonly applied in medical image analysis. However, the success of these approaches is highly dependent on the availability of large manually detailed annotated dataset. Thus an automatic refined segmentation of whole-slide image (WSI) is significant to alleviate the annotation workload of pathologists. But most of the current ways can only output a rough prediction of lesion areas and consume much time in each slide. In this paper, we propose a fast and refined cancer regions segmentation framework v3_DCNN, which first preselects tumor regions using a classification model Inception-v3 and then employs a semantic segmentation model DCNN for refined segmentation. Our framework can generate a dense likelihood heatmap with the 1/8 side of original WSI in 11.5 minutes on the Camelyon16 dataset, which saves more than one hour for each WSI compared with the initial DCNN model. Experimental results show that our approach achieves a higher FROC score 83.5% with the champion’s method of Camelyon16 challenge 80.7%. Based on v3 DCNN model, we further automatically produce heatmap of WSI and extract polygons of lesion regions for doctors, which is very helpful for their pathological diagnosis, detailed annotation and thus contributes to developing a more powerful deep learning model.

## Introduction

Breast cancer is a common disease among women, which is responsible for about 15% of all new cancer cases in the U.S.A, 2018^1^. But its diagnosis often involves the detection of tumor regions from a huge histopathologic whole slide image (WSI), which can be extremely time-consuming and error-prone. To assist in this examination, more and more researchers focus on automated detection of cancer metastases in the WSI. Most of the current methods just concentrate on the rough localization of lesion areas and producing low-resolution probability heatmap. However, in this paper, we emphasize more on refined segmentation of WSIs. This is because refined segmentation has more benefits. Firstly, now supervised learning approaches are still mainstream in medical image analysis. But the success of these methods depends on the availability of a large amount of high-quality manual annotations. It is obvious that refined segmentation can better help doctors label huge whole slide images than those rough segmentation schemes. Secondly, refined segmentation also makes it easier for the subsequent grading algorithm to extract reliable features from finer heatmaps. Thirdly, pathologists can also grade breast cancer more accurately if given better segmentation results.

However, it is not a simple task to process whole-slide images (WSIs) like nature images. Typically, WSIs contain many data layers and there can be billions of pixels, such as 100,000 × 100,000 at the highest resolution, such as 40×, which makes it more complex and time-costing to perform detection on such huge images. Many researchers only analyzed small histopathological images sampled from certain lesion regions of WSI^2–4^ and evaluated their strategies on some small datasets and in low resolution, which are not robust in the real application.

Recently, due to the advancement of the high-resolution scanner and computer technology, more researchers begin to assess their algorithms on WSI datasets. For example, Bejnordi *et al*.^5^ detected the ductal carcinoma *in situ* (DCIS) in WSIs using a multiscale superpixel segmentation method. Balazsi *et al*.^6^ realized a system to detect invasive ductal breast carcinoma. They firstly segmented the WSI by superpixel method in low resolution and then classified every superpixel region into health or cancer tissue using random forest classifier.

Besides the aforementioned traditional image processing and machine learning approach, deep learning is also applied to WSIs. On the task of invasive breast carcinoma detection, CruzrRoa *et al*.^7^ used a simple convolutional neural network (CNN) with three layers to detect invasive breast carcinoma in WSIs on 2× resolution, which made 4% and 6% improvement compared with traditional methods using feature extraction. However, the above methods are evaluated on their own small datasets.

In 2016, IEEE International Symposium on Biomedical Imaging (ISBI) organized Camelyon16 Challenge^8^ to detect cancer metastasis in lymph node and provided 400 WSIs with detailed annotations for training and testing. Different from the natural image, WSI can’t be used for training the neural network directly because of containing billions of pixels. Most of the current methods^9–11^ train deep neural networks on patch images extracted from WSIs at a certain level and they obtained the best performance at 40×. Wang *et al*.^10^ finally won this competition. They firstly cut WSIs into lots of region images with the size of 256 × 256 named patches and trained a GoogLeNet^12^ classification model using these patches to detect cancer regions. They also carried out experiments using patches at different resolutions respectively, such as 10×, 20×, and 40×, and the experiments show that patches at the largest resolution 40× got the best results. On the same dataset, Liu *et al*.^9^ trained an Inception-v3^13^ classification model based on 299 × 299 patches and improved the metric of cancer location. Finally, they produced a low-resolution prediction heatmap with the 1/128 side of original WSI.

However, these existing classification methods have some limits. Firstly, patches extracted with different sizes contain different contextual information, but these models are all trained based on small patches which leads to the contextual information loss. Secondly, each slide includes 10,000 to 400,000 patches (median 90,000)^9^, which makes these ways very time-consuming. Thirdly, these classification models can only create a low-resolution prediction instead of refined segmentation because thousands of pixels in one patch images are given only one label, such as Liu’s work^9^. If they want to produce a denser prediction, like a heatmap with 1/8 side of original WSIs, the test stride should be decreased from 128 pixels to 8 pixels. So the number of patch images and processing time for inference will be increased from *N* to 256 × *N*. It’s sharply time-consuming and unpractical in the real application.

Different from classification methods, semantic segmentation network can classify every pixel in the patch into tumor or normal instead of classifying the whole patch image into tumor or normal. Due to pooling layers, the output prediction will be downsampled. And the sampling rate will be decreased as the network architecture goes deeper. In order to maintain a dense output, Chen *et al*.^14^ proposed a semantic segmentation framework, dense deep convolutional neural network (DCNN), which utilized an atrous convolution operation to replace traditional convolution and pooling operation. It can generate dense prediction and maintain the same receptive field by upsampling operations. But performing semantic segmentation on WSI can spend much time and memories for processing billions of pixels. Besides this, most of the tissues in WSI are normal, where are not necessary to perform semantic segmentation.

In this paper, we propose a cascade framework v3_DCNN for fast and refined breast cancer regions segmentation. Firstly, we apply OTSU segmentation to remove most of the non-tissue background quickly in WSI. v3_DCNN firstly employs a classification model, slimmed-down Inception V3 to rapidly preselect those possible lesion regions in a WSI. Then, a semantic segmentation DCNN model is adopted for refined segmentation. Compared with current methods, our framework can get a dense prediction with the 1/8 side of original WSI in 11.5 minutes, which saves more than one hour for each WSI to produce the same resolution heatmap using semantic segmentation model. Here we give up to produce the heatmap with the same resolution as initial WSI because of the following considerations. (1) In the actual application, we find that the 1/8 side of original WSI is sufficient to assist pathologists in annotation and grading. (2) Upsampling the predictions of 1/8 side to the original size requires quite more time and computer memory, which may be unpractical. Also, we train different models using patches with different contextual information and verify that training model on larger patches can make better use of contextual information and show better overall performance. On Camelyon16 dataset, our framework achieves FROC score 83.5%, which is higher than 80.7% from the champion’s method of Camelyon16 challenge. We also calculate the mIoU metric and Tumor-mIoU metric to measure the result of refined segmentation and the highest Tumor-mIoU metric is 80.69%. Finally, based on v3_DCNN framework, we automatically produce heatmap of WSI and extract polygons of tumor region for the doctors, which is very helpful for pathological diagnosis, computer-aided annotation and thus contributes to developing new deep learning models.

## Camelyon16 Dataset and Evaluation Metrics

To compare with the state-of-the-art strategies, we also evaluate our method on the Camelyon16 dataset^8^, which contains 400 WSIs of lymph node from Radboud University Medical Center and the University Medical Center Utrecht. Table 1 lists the number of slides for training and testing in the Camelyon16 dataset. WSIs are generally stored in a multi-resolution pyramid structure. The largest resolution is 40× magnification, also named level 0. Level 1 is at 20× and the width and height of the image at 20× is a half of that at 40×, and so on. Typically there are seven or eight levels in a WSI.

**Table 1:** Number of slides in Camelyon16 dataset.

Institution	Train (Normal)	Train (Tumor)	Test
Radbound UMC	90	70	80
UMC Utrecht	70	40	50

## Result and Discussion

### Experimental setting

Based on the Camelyon16 test dataset, we design three experiments to justify our proposed method. (1) To analyze the influence of different contextual information on semantic segmentation model, we evaluate our DCNN models using three different training patch size. (2) To illustrate the effectiveness of proposed framework v3_DCNN, we measure the performance of tumor localization and segmentation by several metrics and analyze the running time to produce a dense heatmap. (3) We also evaluate model ensemble among different v3_DCNN models to promote segmentation result. Finally, we compare our method with state-of-the-art methods. To speed up processing time, OTSU segmentation is adopted as a preprocessing stage to remove most of the non-tissue background quickly for each WSI.

### Metrics

In our experiments, besides the aforementioned AUC and FROC metrics, we adopt another metric mIoU (Mean Intersection over Union), which is often used in semantic segmentation, to validate the pixel-level labeling performance.

The way to calculate mIoU is shown as formula (1). The *k* + 1 in formula (1) means the number of classes. The *i* is the label of the groundtruth, and the *j* is the prediction label. The *p*_*ij*_ is the total number of those pixels that are labeled as *j* but predicted as *i*. The mIoU represents the mean of IoUs calculated on both tumor and normal slides. To provide a more reasonable measure on the results of tumor area segmentation, we also employed another metric, Tumor-mIoU, which is the mean of IoUs calculated on tumor slides. All the experiments are carried out just using a single GeForce GTX 1080Ti GPU.

1 $$mIoU=\frac{1}{k+1}\sum _{i=0}^{k}\frac{{p}_{ii}}{{\sum }_{j=0}^{k}{p}_{ij}+{\sum }_{j\mathrm{=0}}^{k}{p}_{ji}-{p}_{ii}}$$

### Evaluation of DCNN models trained with different contextual information

As discussed above, different patch images contain different contextual information, so we train and evaluate our DCNN models with different training patch sizes. We select three patch size of 321 × 321, 768 × 768 and 1280 × 1280 to train our DCNN model respectively. And since semantic segmentation model can adapt to inputs with different sizes during inference, we test our models with 2560 × 2560 patches, the largest size that can be fed into our GPU, to reduce testing time. The AUC and FROC metrics are calculated for comparison. For the AUC calculation, we use the maximum probability of tumor to be the prediction in WSI classification task. In tumor localization task, we use the maximum probability within every lesion region to be the prediction of each region and calculate the FROC metric.

**Figure 1:**
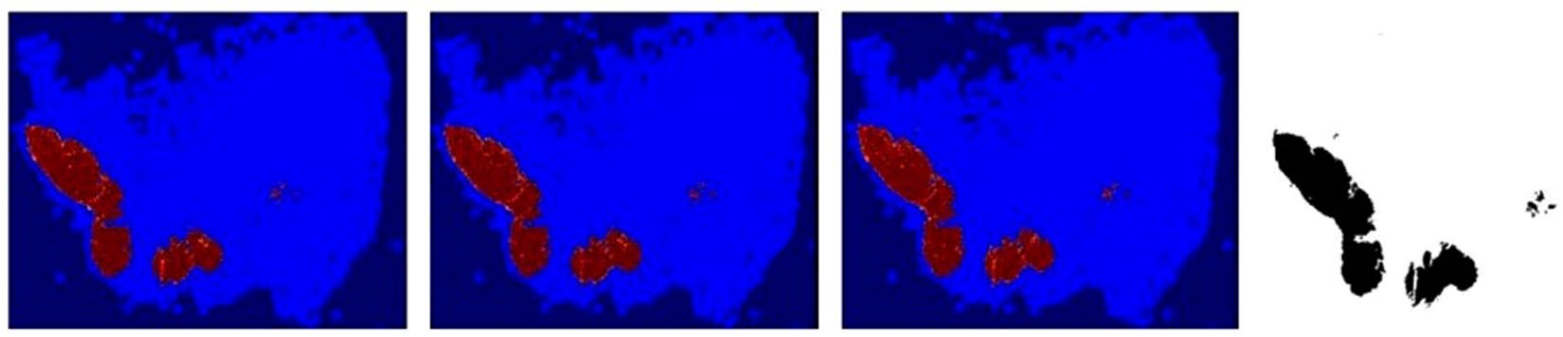
Heatmap from the three models trained on different patches. Redder pixels have the higher probability of being tumor while bluer pixels are more likely to be normal. In Groundtruth, the black regions are tumor and the white areas are normal. From left to right: (**a**) DCNN-321 result (**b**) DCNN-768 result (**c**) DCNN-1280 result (**d**) Groundtruth.

Figure 1 shows the results of three DCNN models based on the different contextual information. Compared with the groundtruth, the heatmaps of the three models cover most of the cancer areas, which verifies the validity of our DCNN model.

For quantitative analysis, we calculate AUC and FROC metrics as Table 2 shows. We also report their 95% confidence intervals using a bootstrap approach: We first random sample slides with replacement from the test dataset and form a new resampled dataset with the same size as the initial test dataset. Then we compute AUC and FROC for this resampled dataset. We repeat these operations for 2000 times and finally report the 2.5% and 97.5% percentile values. The DCNN-1280 model achieves the best result with an overall AUC of 95.0% and an overall FROC of 74.4%, which demonstrates that training utilizing patches with the larger size that containing more contextual information can facilitate tumor localization and segmentation performance.

**Table 2:** AUC and FROC of the three models trained with different contextual information (95% confidence intervals).

Model	AUC (%)	FROC (%)	Heatmap size
DCNN-321	93.6 (89.4, 97.2)	62.8 (51.9, 75.5)	1/8 side of original WSI
DCNN-768	93.4 (88.4, 97.4)	66.5 (55.1, 79.4)	1/8 side of original WSI
DCNN-1280	**95.0** (90.7, 98.3)	**74.4** (63.0, 85.1)	1/8 side of original WSI

### Evaluation of our fast and refined v3_DCNN segmentation framework

In this part, we evaluate our proposed v3_DCNN framework, which adopts a classification model for fast preprocessing and employs DCNN for refined segmenting. Here we directly follow the existing work^9^ and utilize a slimmed-down Inception V3 model for classification.

**Figure 2:**
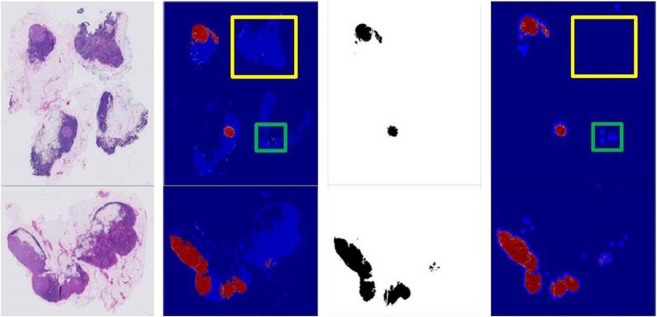
Comparison results of the classification model and our v3_DCNN-1280. From left to right: (**a**) Original image (**b**) Heatmap with 1/128 size (**c**) Groundtruth (**d**) Heatmap with 1/8 side.

As Fig. 2 shows, the classification model can remove most of the normal areas, such as the area inside the yellow rectangle. What’s more, the heatmap produced by the classification model has a low resolution and exists noises. Our segmentation model DCNN can produce a dense prediction which makes the boundary smoother and performs a second filtration on some error detection, such as the normal area in green rectangle.

Table 3 shows the metrics and processing time of different models on the Camelyon16 test set. Our fast segmentation framework v3_DCNN achieve better overall results on all metric than a single classification model and a single segmentation model. The best result was achieved by v3_DCNN-1280 with an overall AUC of 96.6%, an overall FROC of 82.9%, mIoU 68.54% and Tumor-mIoU 80.69%. Through the results of v3_DCNN trained on patches with different sizes, we can also find that the model trained on patches with larger size achieves a better overall result. Larger training patch may contain more contextual information, which will contribute to better segmentation performance. However, note that the performance of our classification model slimmed-down Inception-v3 is lower than the previous work^9^. This may be due to the limitation of our computational resources and detailed parameters selection. We only use one GTX 1080Ti GPU for training while existing method^9^ adopted 8 NVIDIA Pascal GPU in total, which gave an advantage in their model training.

**Table 3:** Comparison of v3_DCNN framework, DCNN-1280 and Inception-v3.

Model	AUC (%)	FROC (%)	mIoU (%)	Tumor-mIoU (%)	Time/slide (min)
DCNN-1280	95.0 (90.7, 98.3)	74.4 (63.0, 85.1)	62.6	79.43	79
Inception-v3	95.8 (90.9, 99.1)	72.9 (63.4, 83.0)	59.05	74.14	6.5^*^
v3_DCNN-321	95.9 (91.5, 99.3)	80.0 (70.8, 88.6)	66.14	79.51	11.5
v3_DCNN-768	95.9 (90.9, 99.2)	82.1 (73.8, 89.4)	64.72	79.88	11.5
v3_DCNN-1280	**96.6** (92.2, 99.6)	**82.9** (75.0, 91.0)	68.54	80.69	11.5

*Inception-v3 takes 6.5 minutes per WSI to generate the heatmap with the 1/128 side of WSI, but the other listed models create the heatmap with the 1/8 side of WSI.

In Table 3, the column Time/slide shows the average processing time for each slide, which includes all the stages, such as preprocessing, extracting patch, model inference and stitching heatmap. To obtain the heatmap with the 1/8 side of WSI, the running time of single segmentation model is 79 minutes, while our v3_DCNN framework only takes 11.5 minutes for each slide. Proposed v3_DCNN can reduce more than one hour for each WSI because the classification model removes most of the normal areas and the number of patches for segmentation is reduced to 6% from 198 K to 12 K. The single classification model Inception-v3 can produce a heatmap with 1/128 side of original WSI by costing 6.5 minutes for each slide. But for a dense heatmap like that with the 1/8 side of WSI, Inception-v3 classification model should reduce the test stride from 128 pixels to 8 pixels, which will sharply increase the number of patches and rise the processing time to about 6.5 × 256 = 1664 min/slide. It is more time-consuming than using a single segmentation model DCNN for the same dense heatmap.

### Evaluation of ensemble models

In order to further improve our segmentation result, we perform the average operation among different v3_DCNN models.

As Table 4 shows, the average of v3_DCNN-768 and v3_DCNN-1280 achieve the best FROC 83.5%. But compared with the model v3_DCNN-1280, the overall AUC, mIoU, and Tumor-mIoU are declined slightly. The best Tumor-mIoU is achieved by v3_DCNN-1280 with 80.69%. It is 6.55% higher compared with 74.14% produced by v3 classification model in Table 3. This shows that our v3_DCNN model can output a more refined segmentation of tumor regions than the classification model.

**Table 4:** Comparison results of ensemble models.

Model	AUC (%)	FROC (%)	mIoU (%)	Tumor-mIoU (%)
v3_DCNN-321 + v3_DCNN-768	95.8 (90.8, 99.5)	81.9 (74.0, 89.5)	66.76	80.11
v3_DCNN-321 + v3_DCNN-1280	96.7 (92.2, 99.6)	82.4 (74.2, 89.6)	67.96	80.18
v3_DCNN-768 + v3_DCNN-1280	96.3 (91.8, 99.5)	**83.5** (75.5, 91.1)	67.76	80.68
v3_DCNN-321 + v3_DCNN-768 + v3_DCNN-1280	96.2 (91.6, 99.5)	83.3 (74.9, 90.5)	64.72	80.39

To evaluate our methods, we compare with the top methods in Camelyon16 challenge^11^ in Table 5. The HMS and MIT won the challenge by combining the results of two GoogLeNet with an overall AUC of 99.4% and an overall FROC of 80.7%. In our methods, the best overall FROC can reach 83.5%, which is comparable with their method. For the AUC metric, HMS and MIT extracted 28 features from the heatmap and trained a random forest classifier to predict the probability of a WSI containing tumors. However, we simply use the maximum probability within a WSI without any complex post-processing for AUC, which may make our AUC metric lower than them. Besides, note that the side of their heatmap is 1/4 of the original WSI. When using a single GTX 1080Ti GPU as ours, the processing time of two GoogleNets will be more than 13000 minutes per slide. However, though our heatmap has a relatively lower resolution, our v3_DCNN achieves comparable performance while using an obviously shorter time. Besides this, compared with pathologists, who requires about 14 minutes per WSI^11^, our proposed v3_DCNN-1280 outperforms these experts in both slide classification and tumor localization while taking about 11.5 minutes per slide.

**Table 5:** Comparison results of our methods and the Camelyon16 methods.

Team	Architecture	AUC (%)	FROC (%)
Pathologist^11^	N/A	96.6 (92.7, 99.8)	72.4 (64.3, 80.4)
HMS and MIT^11^	GoogLeNet	**99.4** (98.3, 99.9)	**80.7** (73.2, 88.9)
HMS and MGH	ResNet	97.6 (94.1, 99.9)	76.0 (69.2, 85.7)
CULab	VGG-16	94.0 (88.8, 98.0)	70.3 (60.5, 79.9)
Our method	v3_DCNN-1280	96.6 (92.2, 99.6)	**82.9** (75.0, 91.0)
Our method	v3_DCNN-768 + v3_DCNN-1280	96.3 (91.8, 99.5)	**83.5** (75.5, 91.1)

Also, we notice that an unpublished approach^9^ in arXiv has reported quite high results in AUC and FROC. When using single slimmed-down Inception-v3 model, their overall AUC and FROC are 97.1% and 86.4% respectively. After applying the model ensemble, the overall AUC and FROC can be improved to 97.7% and 88.5%. Actually, we tried hard to repeat their experiments and wanted to justify the effectiveness of our v3_DCNN based on their Inception-v3 model. But due possible to the restriction on computational resources, our final Inception-v3 model can only achieve 95.8% and 72.9% in the overall AUC and FROC.

**Figure 3:**
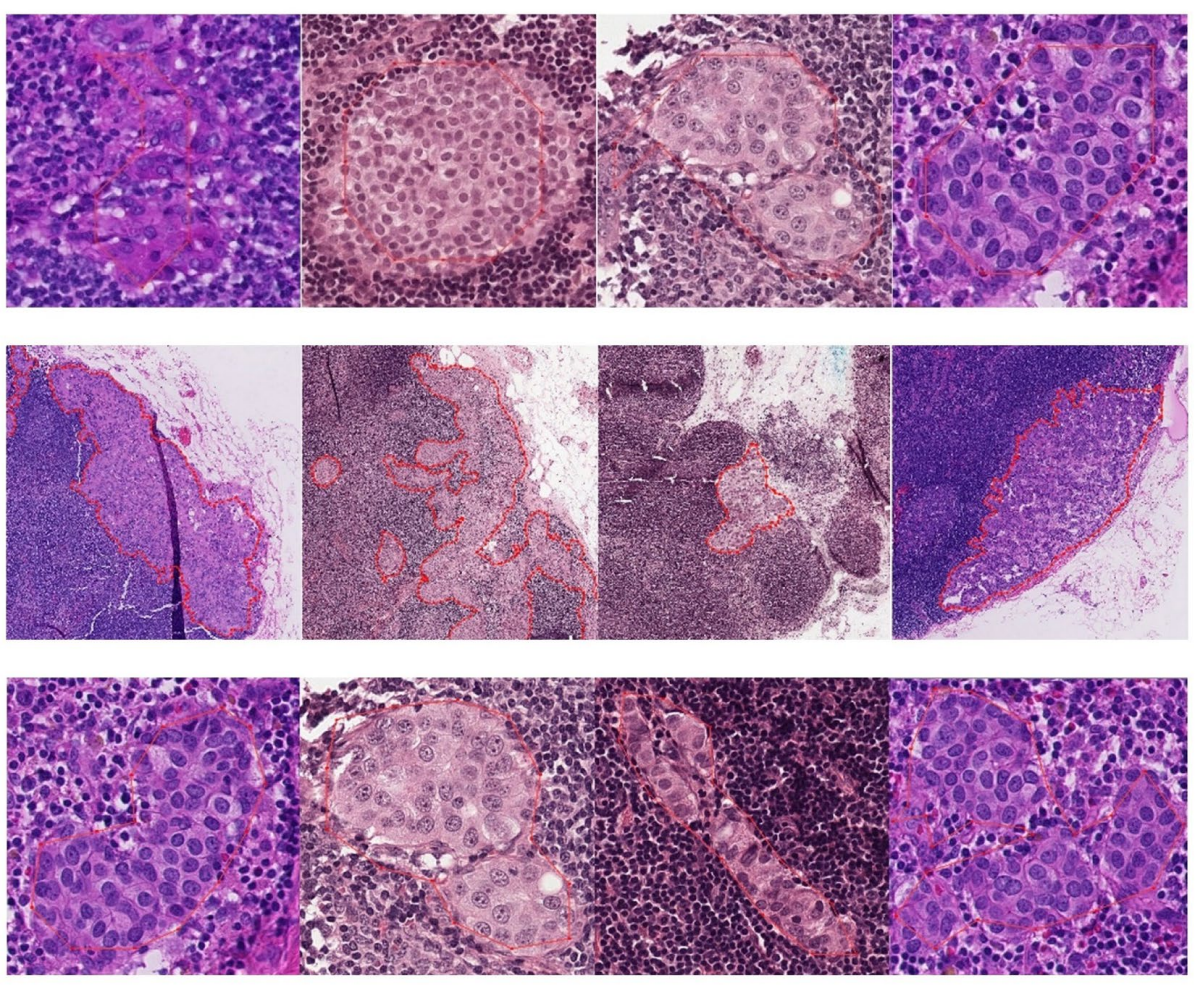
External polygons of tumor regions produced by v3_DCNN-1280 and Inception-v3. From top to bottom are external polygons of tumor regions produced by (**a**) our inception-v3 model shown at 40x (**b**) v3_DCNN-1280 model shown at 5x (**c**) v3_DCNN-1280 model shown at 40x.

Refined segmentation can be also useful for computer-aided annotation on new huge WSI images for doctors. In the real application, the pathologists label tumor regions by drawing the contour with a polygon, which is time-consuming and tedious work. To show our refined segmentation performance, we select the v3_DCNN-1280 model with the highest Tumor-mIoU to produce a binary image from the heatmap using threshold 0.5. And then we adopt a contour retrieving method^15^ to obtain external polygons of all segmented tumor areas. Finally, we draw the polygons using the ASAP open source platform^16^ on the WSIs. As Fig. 3 shows, the first row in Fig. 3 is the external polygons of tumor regions produced by our Inception-v3 model shown at 40×, which is produced based on the heatmap with 1/128 side of WSI by our Inception-v3 model. We can see that it can’t fit the boundary very well. The second row and the third row are the external polygons of tumor regions based on our dense heatmap with 1/8 side of WSI by our v3_DCNN-1280 model in 5× and 40× respectively. Here 5× has equivalent resolution with our dense heatmap with 1/8 side. We can see that the polygons can fit the boundary of tumor areas closely, even at 40×. With our external polygons of tumor regions, doctors can further check or modify the labeling information of tumor regions easily, which will save much time for their labeling work.

## Conclusion

This paper proposes a fast and refined cancer regions segmentation framework in breast pathological WSIs. Firstly, we apply OTSU segmentation to remove most of the non-tissue background quickly. Then we use a simplified Inception-v3 classification model^9,13^ to obtain a rough prediction of tumor areas. We further use our DCNN semantic segmentation model on those preselected areas to get refined segmentation of tumor regions. Experimental results illustrate our proposed v3_DCNN framework can generate a dense heatmap with high localization performance and less processing time, which is important and useful for further tumor diagnosis, grading and computer-aided annotation of huge WSI for pathologists. In the future, we will further improve our tumor segmentation method and test on more breast histopathological images from cooperant hospitals.

## Proposed Method

**Figure 4:**
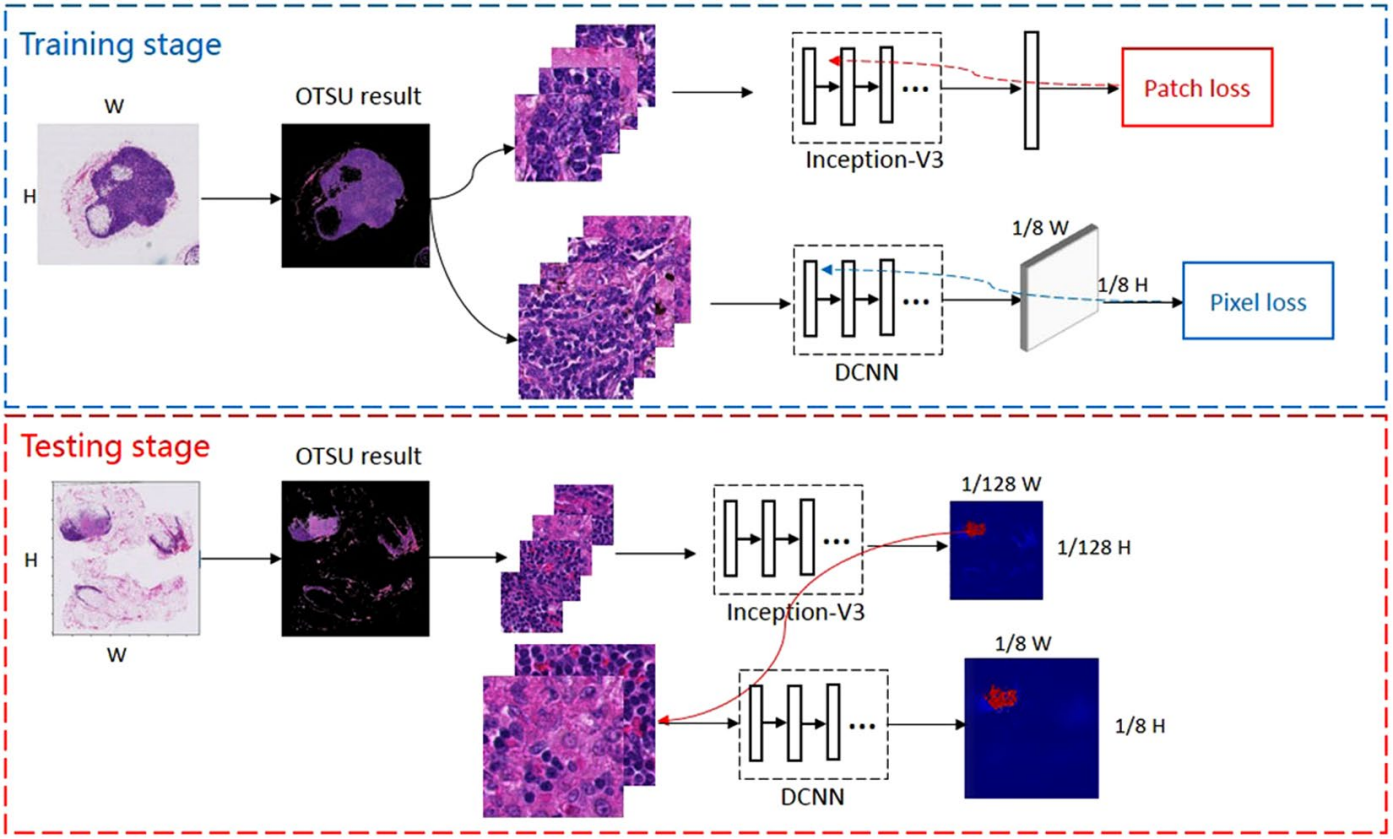
Our fast and refined cancer regions segmentation framework v3_DCNN.

Figure 4 shows our proposed fast and refined cancer regions segmentation framework v3_DCNN. To reduce time, we firstly apply OTSU^17^ segmentation method to remove the non-tissue background in both training and test stages. Then in training stage, we extract patch images from WSI to train classification model slimmed-down Inception-v3^9,13^ and semantic segmentation model DCNN^14^ respectively. The purpose of the classification model is to detect the tumor areas quickly, while the segmentation model is to perform refined segmentation. During testing, we extract small patch to run inference of classification model and then we can get a low-resolution heatmap quickly. Based on this heatmap, we only apply our semantic segmentation model to those regions predicted as tumor by classification model, which can drastically reduce the number of testing patches and save much time for segmentation. The details will be introduced in followings.

### Preprocessing of WSI by Removing Non-tissue Background

**Figure 5:**
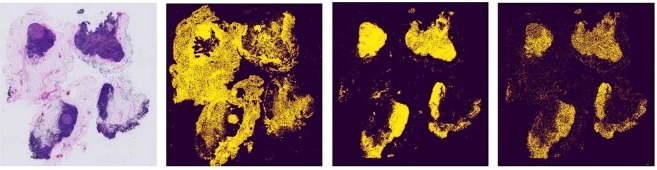
Image preprocessing results using OTSU segmentation. The yellow pixels are the foreground and the purple pixels are the background. From left to right: (**a**) Original image (**b**) OTSU on H channel (**c**) OTSU on S channel (**d**) OTSU on V channel.

As Fig. 5 shows, a typical WSI contains many non-tissue backgrounds. We filter out the background using a quick segmentation method OTSU^17^. It is unreasonable to apply OTSU directly to the WSI at 40× magnification, which contains about 100,000 × 100,000 pixels. So we employ OTSU on lower 1.25× magnification. Then we map the position of the foreground in the filtered slide to the WSI at 40× magnification for patch extraction. Specially speaking, we first convert RGB to HSV color space and then apply OTSU on H, S and V three channels respectively. The experiment shows that the best result was achieved on the S channel as Fig. 5 shows. Using this preprocessing, about 82% background region will be removed, which will be used on both training and test stages.

### Cancer Regions Refined Segmentation by Semantic Segmentation Model

As discussed in the introduction, existing methods with classification models can only produce a rough prediction instead of refined segmentation. Decreasing the test stride of inference will extremely increase the number of patches and processing time. We adopt semantic segmentation model to realize refined segmentation of cancer regions. We improve the DCNN^14^ model by using Resnet-101^18^ architecture as our segmentation model. In our model, most of the traditional convolution layers and pooling layers are replaced by atrous convolution. Considering computation ability and memory of a general computer, we abandon the bilinear interpolation operation in the model and produce a dense output with 1/8 side of original 40× WSI.

Different from the classification model using patch loss, our DCNN model use pixel loss to calculate loss for gradient backpropagation. Assumed that the size of input patches is *L* × *L*, *p* (*x*, *y*) represents that the probability that the pixel at coordinate (*x*, *y*) is a tumor pixel in prediction image, where 0 ≤ *x* < *L*/8 and 0 ≤ *y* < *L*/8, *m* represents the corresponding mask of input patch, where the tumor is labeled with 1. So we can calculate the pixel loss of a patch image by the following formula.

2 $$pixel\,loss=-\,\frac{1}{{(\frac{L}{8})}^{2}}\sum _{x=0}^{L\mathrm{/8}}\sum _{y=0}^{L\mathrm{/8}}[\,\mathrm{ln}\,p{(x,y)}^{m\mathrm{(8}x\mathrm{,8}y)}+\,\mathrm{ln}\,{(1-p(x,y))}^{1-m\mathrm{(8}x\mathrm{,8}y)}]$$

After testing all patch images from one WSI, we stitch all patch predictions to a whole heatmap, which is as large as the image in level 3, at 5× magnification. In detail, we will record the top left coordinate of every patch in level 0 image as (*x*_*i*_, *y*_*i*_). After inference, the size of output prediction is 1/8 side of the original patch. So the coordinate of output prediction of every patch in level 3 image is (*x*_*i*_/8, *y*_*i*_/8). Since there are some overlaps of patches, we take the average of the probability of all predictions at the same coordinate. So the probability at the coordinate (*x*, *y*) in heatmap can be calculated by following formula.

3 $${p}_{h}(x,y)=\frac{1}{n}\sum p({x}_{i}\mathrm{/8,}{y}_{i}\mathrm{/8)},\,for\,{x}_{i}/8=x\,and\,{y}_{i}/8=y$$

where *n* represents the number of the coordinates satisfying the condition *x*_*i*_/8 = *x*&&*y*_*i*_/8 = *y*.

Our DCNN model can produce a denser heatmap with 1/8 side of WSI using less time than classification model with the same heatmap size. However, most WSIs are normal slides in a real application and even in tumor WSI, most of the tissues are normal, which are unnecessary for refined semantic segmentation. So if we can filter out most of the normal regions quickly, our DCNN model can be speeded up greatly.

### A Fast and Refined Cancer Regions Segmentation Framework v3_DCNN

Based on above-mentioned advantages of the classification model and semantic segmentation model, we propose a fast and refined cancer regions segmentation framework v3_DCNN based on the preselection of tumor regions using classification model and the further refined segmentation results utilizing our DCNN model.

As Fig. 4 shows, we firstly apply OTSU method to remove most of the non-tissue background in a WSI. Then we sample small patches to run inference of classification model for selecting the candidate tumor areas and producing a rough heatmap quickly. Based on this rough heatmap, we separate the tumor areas predicted by classification model with a threshold 0.5 and extract large patches centered on the position of candidate tumor pixel from the WSIs at 40× for refined segmentation. Finally, we stitch the output predictions according to the formula (3) to create a dense heatmap. The details are introduced as follows.

#### Classification model

We adopt slimmed-down Inception-v3 architecture^9,13^ to be our classification model. Compared with the GoogLeNet used in Wang’s^10^ paper, an important improvement of Inception-v3 is factorizing symmetric convolution into small asymmetric convolutions, such as using a 3 × 1 convolution followed by a 1 × 3 convolution to replace a 3 × 3 convolution. The spatial factorization can reduce much processing time, enhance the nonlinearity and increase the depth of the neural network, which is very suitable for preselection of tumor areas in our v3_DCNN framework. To further reduce computing time of Inception-v3, we also cut down the number of convolutional filters and only remain the 10% of the original model. So this slimmed-down Inception-v3 model has less calculation complexity and can preselect tumor areas faster.

**Figure 6:**
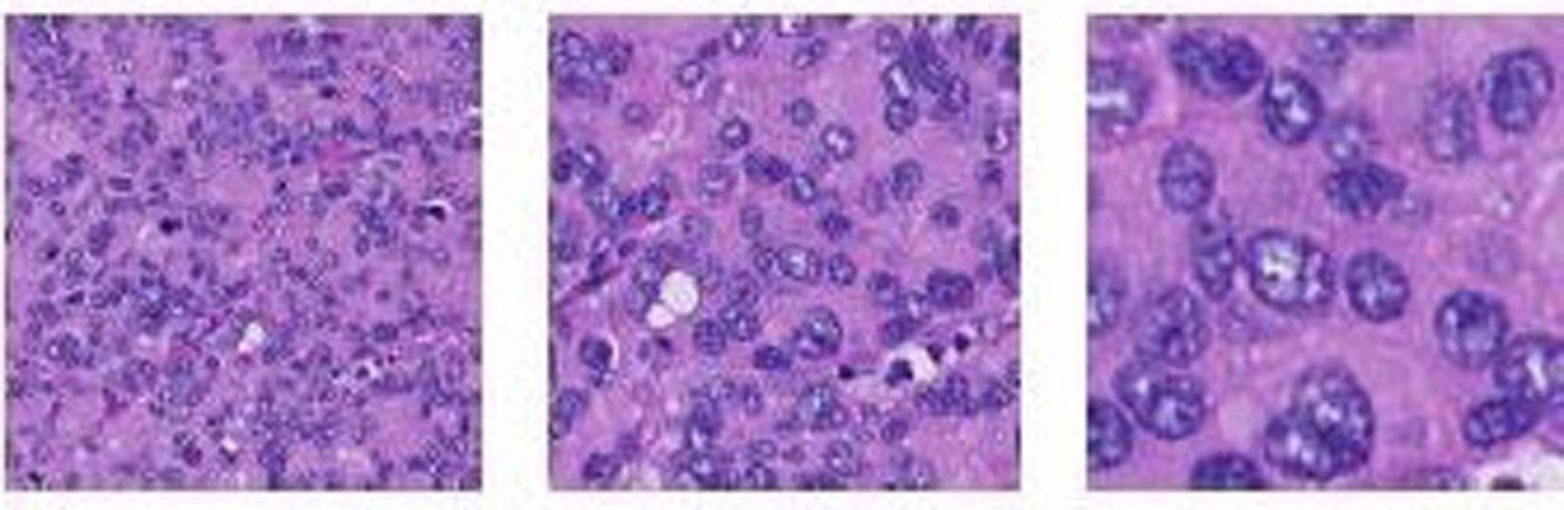
The patch images with the same size at different magnification. From left to right: (**a**) 10× (**b**) 20× (**c**) 30×.

Different from the natural image, WSI can’t be used for training the neural network directly because of containing billions of pixels. Most of the current methods^9–11^ train deep neural networks on patch images extracted from WSIs at a certain level and they obtained the best performance at 40×. As Fig. 6 shows, the patch image at a lower magnification contains a larger receptive field, but it suffers from more cell-level information loss. Our classification model uses patch images with 299 × 299 size (the default size of Inception-v3) at 40× to extract features of cells more effectively.

Implementation details: In the training stage, we first remove the non-tissue background by OTSU segmentation on the Camelyon16 training set. And then we extract 299 × 299 patch images from the rest tissues and label them as tumor or normal according to their groundtruth. A patch image located in a tumor region is treated as a tumor patch and labeled with 1. Assumed that the probability of the patch containing tumor pixel is *p*, the label is *g*, *n* represents the number of patches in a batch. So the patch loss can be calculated by the following formula.

4 $$patch\,loss=-\,\frac{1}{n}\sum _{i=1}^{n}\,[\mathrm{ln}\,{p}_{i}^{{g}_{i}}+\,\mathrm{ln}\,{\mathrm{(1}-{p}_{i})}^{1-{g}_{i}}]$$

Since our Inception-v3 model has less calculation and can be iterated quickly during training, we use all the patch images extracted from the training set to train our classification model. To enrich the diversity of training samples, we also apply the usual data augmentation operation, such as mirror flip, rotation, color jittering, to samples when training. In the test stage, we apply OTSU firstly and then extract 299 × 299 patch images at 40× with a stride 128 pixels from the test WSIs. After testing all patches from a WSI, we stitch all the output predictions according to the formula (3) and produce a low-resolution heatmap with 1/128 side of WSI in final.

#### Semantic segmentation model

**Figure 7:**
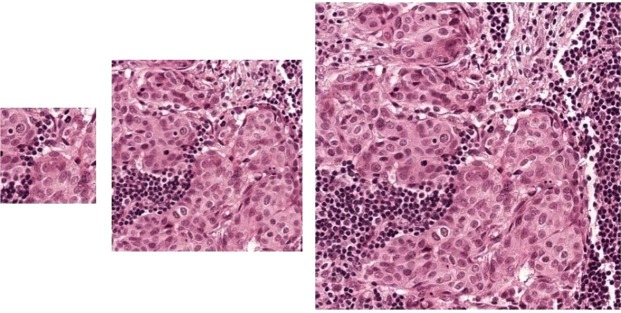
The patch images with different sizes at 40× magnification. From left to right: (**a**) 321 × 321 (**b**) 768 × 768 (**c**) 1280 × 1280.

Different from the classification model to produce a rough prediction quickly, the goal of our semantic segmentation model DCNN is to obtain the refined segmentation of tumor regions. But training model on small patch images will lose lots of contextual information. Obviously, the patch image with a larger size contains more contextual information. To our best knowledge, when pathologists diagnose breast cancer, they will not only check the characteristics of cells but also observe the tissue around. It may be difficult for the pathologists to find out the cancer region just through observing a small patch image. So we believe that different patch images containing different contextual information will have a different impact on model training and testing. We sample patches with different sizes at 40× to train our DCNN model including 321 × 321, 768 × 768 and 1280 × 1280. As Fig. 7 shows, the larger patch at 40× contains larger context and contains detailed cell information.

**Figure 8:**
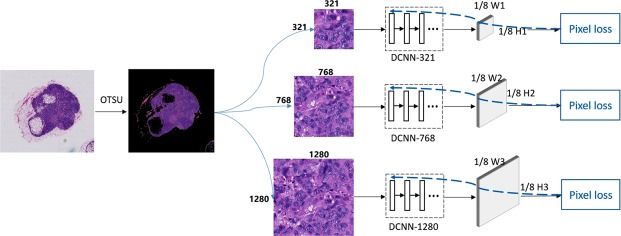
Training DCNN models by using different patch images.

Implementation details: Figure 8 shows our DCNN training framework. We train three DCNN models independently with different patch images. According to the sizes of training patches, we name three DCNN models as DCNN-321, DCNN-768, and DCNN-1280 respectively. In the training stage, we firstly filter out the background by OTSU method and then extract patches. To accelerate the training process, we select normal patch images randomly instead of using all of them and make the rate between normal and tumor is 1:1. We also implement the data augmentation operation, such as mirror flip, rotation, color jittering, to enrich the diversity of training samples. Different from the classification model using patch loss, we need to calculate the pixel loss for gradient backpropagation of semantic segmentation model. Since the output of our DCNN model will be downsampled with a 1/8 sampling rate, we downsample the patch label mask to calculate the loss as formula (2) shows.

Inference of our cascade framework v3_DCNN: In the test stage, we separate the tumor areas predicted by classification model with a threshold 0.5 and extract large patch images centered on the position of tumor pixel from the WSIs at 40× for refined segmentation. Because our segmentation model can adapt to inputs of different sizes, we extract patch image as large as possible to decrease the number of test patches and reduce processing time. In our experiments, the size of the test patches for the three models is the same 2560 × 2560. We also make every two adjacent patches contain a half overlap in order to improve the robustness of our segmentation model. After testing all patches in a WSI, we stitch all the patch heatmaps into a whole-slide heatmap. The side of the dense heatmap is 1/8 of original WSI, which equivalent to the size of the image in level 3.

## Data Availability

The data used in this study was waived review and consent by the institutional review board. All data was being anonymized. The dataset is available in the Camelyon16 challenge [https://camelyon16.grand-challenge.org].
