# Publisher Correction: A Fast and Refined Cancer Regions Segmentation Framework in Whole-slide Breast Pathological Images

**DOI:** 10.1038/s41598-020-65026-9

**Published:** 2020-05-20

**Authors:** Zichao Guo, Hong Liu, Haomiao Ni, Xiangdong Wang, Mingming Su, Wei Guo, Kuansong Wang, Taijiao Jiang, Yueliang Qian

**Affiliations:** 1 Beijing Key Laboratory of Mobile Computing and Pervasive Device, Institute of Computing Technology, Chinese Academy of Sciences, Beijing, 100190 China; 2 Research Center for Big Data of Biomedical Sciences, Institute of Basic Medical Sciences, Chinese Academy of Medical Sciences & Peking Union Medical College, Beijing, 100005 China; 3 Suzhou Institute of Systems Medicine, Suzhou, 215123 China; 4 Graduate School of Peking Union Medical College, Beijing, 100005 China; 5 Department of Pathology, Xiangya Hospital, Central South University, Changsha, Hunan 410013 China; 6 Department of Pathology, School of Basic Medical Sciences, Central South University, Changsha, Hunan 410013 China

Correction to: *Scientific Reports* 10.1038/s41598-018-37492-9, published online 29 January 2019

In the original version of this Article, Yueliang Qian was incorrectly listed as a corresponding author. The correct corresponding authors for this Article are Hong Liu, Kuansong Wang and Taijiao Jiang. Correspondence and request for materials should be addressed to H.L. (email: hliu@ict.ac.cn) or K.W. (email: 13787146109@126.com) or T.J. (email: taijiao@ibms.pumc.edu.cn).

This error has now been corrected in the HTML and PDF versions of the Article.

